# The Translational Potential of Electrochemical DNA-Based Liquid Biopsy

**DOI:** 10.3389/fchem.2020.00143

**Published:** 2020-03-20

**Authors:** Rebeca Miranda-Castro, Ilaria Palchetti, Noemí de-los-Santos-Álvarez

**Affiliations:** ^1^Departamento Química Física y Analítica, Universidad de Oviedo, Oviedo, Spain; ^2^Instituto de Investigación Sanitaria del Principado de Asturias, Oviedo, Spain; ^3^Dipartimento di Chimica Ugo Schiff, Università degli Studi di Firenze, Florence, Italy

**Keywords:** cancer, liquid biopsy, nucleic acids, electrochemical biosensors, ctDNA, exosomes, CTCs, aptamer

## Abstract

Latest technological advancement has tremendously expanded the knowledge on the composition of body fluids and the cancer-associated changes, which has fueled the replacement of invasive biopsies with liquid biopsies by using appropriate specific receptors. DNA emerges as a versatile analytical reagent in electrochemical devices for hybridization-based or aptamer-based recognition of all kind of biomarkers. In this mini review, we briefly introduce the current affordable targets (tumor-derived nucleic acids, circulating tumor cells and exosomes) in body fluids, and then we provide an overview of selected electrochemical methods already applied in clinical samples by dividing them into three large categories according to sample type: red (blood), yellow (urine), and white (saliva and sweat) diagnostics. This review focuses on the hurdles of the complex matrices rather than a comprehensive and detailed revision of the format schemes of DNA-based electrochemical sensing. This diverse perspective compiles some challenges that are often forgotten and critically underlines real sample analysis or clinical validation assays. Finally, the needs and trends to reach the market are briefly outlined.

## Introduction

Only 30 years after the discovery of circulating tumor DNA (ctDNA) as a type of circulating free DNA (cfDNA) (Stroun et al., 1989) the dream of interrogating the dynamic molecular profile of solid tumors from a blood test is being enthusiastically pursued. Liquid biopsy, the detection of tumor or tumor-derived material in body fluids, has emerged as a minimally invasive subrogate of tissue biopsy. Body fluids are complex matrices containing representative molecules that correlate with the pathological status and progression of disease. The ultimate goal is early detection of cancer and its recurrence through longitudinal studies that allow better patient stratification, but also to observe the response to therapy and drug-resistance earlier than clinical and imaging diagnostics (Crowley et al., 2013; Keller and Pantel, 2019). This attractive concept has bloomed in less than a decade to be integrated in cancer management, which would be impossible without extremely sensitive and selective analytical methodology and the recently available bioinformatic tools.

Besides circulating tumor cells (CTCs), tumor materials released to biological fluids comprise of cell products, mainly nucleic acids (NA), and extracellular vesicles (EV). High throughput and genomic wide analysis of these biomarkers requires sophisticated instrumentation and handling huge amounts of data. Though invaluable, implementation of these technologies is not straightforward, especially in low-resources settings. To reduce the elevated expenditure of health systems in *in-vitro* diagnostics, biosensors-based devices have appeared as a point-of-care (POC), decentralized, rapid, and low-cost alternative. Among them, electrochemical platforms—the focus of this perspective—are emerging in the clinical field due to miniaturization and multianalyte detection capability in untreated body fluids. Smart measurement strategies provide real-time signal drift correction and adjusted time-resolution, allowing for continuous therapeutic drug monitoring (Aller Pellitero et al., 2019). These promising applications based on self-reporting DNA receptors have not yet been applied to humans or to detect tumor biomarkers.

**Figure 1:**
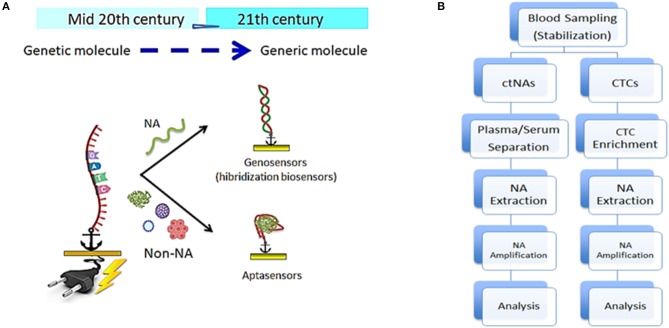
**(A)** Versatility of DNA-based sensors schematics; genosensors based on hybridization reaction to detect any nucleic acid and aptasensors based on conformation and chemical affinity to detect non-nucleic acid analytes. **(B)** Comparative step by step pre-analytical workflow for the analysis of clinical blood samples to detect circulating tumor nucleic acid (left) and circulating tumor cells (right).

It is fascinating that the analysis of known NA point mutations and the detection of cells, EV, and their cargo can be accomplished using a single type of probe: DNA, the most versatile molecule (Figure 1A). It recognizes either NA through complementary hybridization or protein biomarkers on EV and CTCs surface through specific affinity interactions (aptamer-based detection), similarly to antigen-antibody recognition. Herein, after a description of the main targets and their challenges as biomarkers, we focus on selected methods applied to blood (red diagnostics), urine (yellow diagnostics), and saliva and sweat (white diagnostics) following the requirements for *in-vitro* diagnostics with POC devices.

## Targets in Liquid Biopsies

Correlation between levels of CTCs or ctDNA and disease progression/remission/recurrence is the rationale behind their potential use in guiding cancer care. However, their extremely low amount at early disease stages and the high prevalence of tumor-associated mutations in a healthy aged population imposes a boundary to apply them as early-detection biomarkers (Keller and Pantel, 2019).

CtDNA appears in body fluids as even shorter fragments than cfDNA (<200 bp), though there are longer strands that are poorly recovered by current extraction methods (Wan et al., 2017). The short length facilitates the hybridization reaction on biosensor surface and their half-life, <2.5 h, provides a real-time picture of the disease stage and evolution but also imposes challenges in sample collection that needs to be standardized (Rossi and Ignatiadis, 2019). The ctDNA-to-cfDNA ratio varies on disease stage and tumor type (0.1–5%) (Keller and Pantel, 2019), which means the cfDNA is much more abundant than the target complicating the detection. Most cfRNA in body fluids are non-coding RNA and mRNA. Unlike ctDNA the mRNA copy number is larger than two, up to thousands copies, so their concentration in fluids is higher (Siravegna et al., 2017). Non-coding RNAs, formerly known as dark matter, are now regarded as crucial molecules in cancer biology (Miranda-Castro et al., 2019). The shorter RNAs (mi-RNA) have been studied more intensively than the longer counterparts (lncRNA) but both are promising markers in spite of the presence of ribonucleases because they used to be encapsulated in EV or linked to other macromolecules (Wang et al., 2019a).

Beyond free NA, other structures carrying tumor-related components such as aberrant proteins or NAs are regarded as promising biomarkers. CTCs presence in body fluids is associated to poor prognosis and recurrence. Their isolation from an ocean of blood cells is challenging, though not always required. Antigen-dependent enrichment uses antibodies against cell surface markers, but these signatures can be lost during malignant transformation. Cocktails of antibodies could alleviate this issue (Siravegna et al., 2017). As an alternative, cell-SELEX provides a convenient tool to select DNA-based receptors customized to the aberrant features of tumor cells and there are already commercial kits using specific aptamers for this purpose.

EVs are membranous structures secreted by several tissues through different mechanisms originating three distinct vesicle types. Of these, exosomes stand out because their cargo (cfNA and proteins) might mirror the cell of origin. For analytical purposes, exosome isolation through antigen-dependent methods that can be integrated in analytical platforms are preferable over physical methods that are technically demanding and cannot discriminate between EV subtypes containing more relevant information (Wang et al., 2019a).

The nucleic acid nature of capturing aptamers provides an easy way of recovering intact CTCs or exosomes for downstream applications (Zhang et al., 2019). Besides isolation, specific CTC and exosome detection using aptamers is an attractive alternative to immunoassays under active investigation.

## DNA-Based Red Diagnostics

Smart and very sensitive (fM and aM) electrochemical strategies have been proposed for the determination of NAs (Bettazzi and Palchetti, 2018; Tavallaie et al., 2018; Das and Kelley, 2019; Soda et al., 2019). However, to the best of our knowledge, so far no examples of electrochemical DNA-based platforms have been proposed for the analysis of ctNAs in untreated human blood samples, just after venipuncture. Thus, electrochemical red diagnostics for ctNA monitoring faces the same sample pre-analytical issues as the ones reported for other analytical techniques. In fact, monitoring of ctNAs is generally performed by using pre-analytical steps consisting of blood collection, sample stabilization, sample processing, NA extraction, and amplification. A schematic of a possible multistep workflow is reported in Figure 1B, including an additional cell enrichment step followed by NA extraction or amplification by cell lysis when the target is NA contained in CTCs.

Blood collection is mainly performed in collection vessels containing EDTA where leucocyte lysis occurs, leading to cellular genomic contamination; therefore, stabilizing blood collection is important in obtaining reproducible and sensitive analytical data. Nowadays, collection devices with preservative reagents are commercially available, and some recent reports review the performance of these collection devices (Toro et al., 2015; Salvianti et al., 2019). Blood storage conditions are another crucial step that should be carefully designed, since degradation and fragmentation of ctDNAs is possible during storage. Blood processing is mainly performed by centrifugation, and procedures have been developed to obtain reproducible results (i.e., double centrifugation steps at known velocity and time) and to obtain stable plasma and serum samples. Owing to the low abundance of ctNAs, NA extraction and amplification are generally performed. Both manual and automated extraction procedures are currently in use, with commercially available extraction kits. These kits operate via liquid-phase isolation methods, solid-phase extraction methods based on silica gel spin columns, and magnetic beads based separation methods. In comparison to ctDNAs, ctRNAs are more fragile and easily prone to degradation due to the presence of ubiquitous RNAses, however, similar protocols can also be applied for RNA extraction and analysis (Haentzsch et al., 2014; El-Khoury et al., 2016).

Many gold standard techniques for ctNA monitoring require a sample-amplification step, generally performed by PCR. Biosensing platforms hold great promise for a simple and rapid detection of ctNAs, since they are claimed to skip the time-consuming extraction and PCR amplification step, and thus, allow for the possibility to be used as screening tests for POC analysis (Finotti et al., 2018). Nano-structuration of the electrochemical platforms, obtaining improved surfaces in terms of higher electroactive surface area and oriented capture probe immobilization (Voccia et al., 2017; Ingrosso et al., 2019), and the development of reliable signal-amplification techniques are important factors to explain the improved detection performance of the electrochemical devices (Voccia et al., 2016). Plasma and serum are the matrices where electrochemical platforms are mainly tested. A PCR-free biosensing approach capable of detecting KRAS and BRAF mutations in the serum of patients with lung cancer and melanoma with an electrochemical clamp assay was proposed by Kelley's group (Das et al., 2015). The clamp chip detected mutated sequences of both ctDNAs and ctRNAs directly in the patient serum, without any previous extraction procedure. The serum sample was mixed with many oligonucleotide sequences that sequester the wild-type sequence and all the mutants except the detection target. The sample was then applied to a PNA probe-modified nanostructured chip, and only the target mutant hybridizes to the immobilized PNA capture probes. The other mutants and wild-type nucleic acids are prevented from binding. Differential pulse voltammetry, using an electrocatalytic reporter system (i.e., Ru(NH_3_)_6_^3+^ and Fe(CN)_6_^3−^), is used for monitoring the hybridization reaction. The same group reported a similar approach for the monitoring of all 40 somatic mutations of the EGFR gene directly in patient serum by using a nanostructured electrochemical chip modified with 7 capture probes (Das et al., 2018).

Enzymes are also frequently used to improve the signal amplification in a PCR-free analytical scheme (Bettazzi et al., 2013; Voccia et al., 2016). Recently, a PCR-free electrochemical approach has been reported that detects the most frequent DNA methylation markers (5-methylcytosine, 5-mC, and/or 5-hydroxymethylcytosine, 5-hmC) directly in serum samples from cancer patients (Povedano et al., 2019).

Analysis of plasma samples other than serum samples is also quite frequently performed. Recently, extremely low amounts of ctDNA (KRAS G12DM) have been achieved in plasma with a label-free biosensor based on double amplification strategy (namely target recycling and isothermal non-templated DNA elongation), though it still requires previous ctDNA extraction (Wang et al., 2018). Cancer-associated epigenetic modifications such as cytosine methylation leading to changes in DNA properties on Au surfaces were also explored in order to detect cancerous genomes in treated plasma samples (Sina et al., 2018).

Regarding the analysis of NAs present in CTCs, an analytical platform integrating electrical lysis and release of cellular targets, isothermal NA amplification and nanozyme-mediated electrochemical detection has been proposed by Trau's group (Koo et al., 2018).

The detection of specific features of CTCs (aberrant protein expression) or tumor-specific protein released into the bloodstream has benefited from the search for novel therapeutic aptamers. Magnetic particles functionalized with aptamers allows for the decoupling of biorecognition and electrochemical transduction, thus minimizing electrode biofouling. This effect could be monitored through for example, the enzymatic hydrolysis of a nitrocellulose film covering the electrode (Malecka et al., 2019), or the on-surface hybridization of a DNA probe acting as a surrogate for the protein biomarker (Liu et al., 2019). Even so, analysis of diluted serum samples is still required, thus demanding more sensitive approaches to detect clinically relevant protein levels. Aptamer-based assays challenged to clinical serum samples (from healthy individuals and cancer patients) showed satisfactory agreement with the immunomethod routinely used in hospitals (Wen et al., 2016; Ma et al., 2018a; Liu et al., 2019; Wang et al., 2019b). Nevertheless, their complexity in steps and reagents (Wen et al., 2016; Ma et al., 2018a; Liu et al., 2019) could discourage their implementation in clinical practice. Of greater simplicity are signal-off tests (Wang et al., 2019b), although they require very strict selectivity controls.

The obvious replacement of antibodies with aptamers to detect the few cancer biomarkers in clinical usage cannot improve the limited clinical utility of those biomarkers. Accumulated evidence shows that novel approaches targeting abnormal glycosylation of proteins among other posttranslational modifications associated to tumorigenesis can be more informative than the total amount of a biomarker (Díaz-Fernández et al., 2018). These strategies are feasible by rational selection of aptamers as recently reported for the detection of PSA in clinical serum samples with a sandwich-type aptasensor. Combining a protein-binding anti-PSA aptamer with an aptamer directed to the glycan moiety enhanced specificity compared to commercial ELISA was achieved when considering final medical diagnosis (Díaz-Fernández et al., 2019).

Regarding the numerous aptasensors for cancer cells, the great performance of nanomaterials is a common feature (Sun et al., 2019) that was brought to light with multiplexing in whole blood (Dou et al., 2019). Interestingly, the release of viable CTCs for downstream analyses has been accomplished by an enzymatic digestion step of a uracil-containing aptamer sequence partially hybridized with a complementary sequence attached to the electrode. The resulting faradaic impedance cytosensor is reusable up to eight times (Shen et al., 2016). An attractive feature of aptamers is the possibility of exploiting DNA amplification techniques to improve sensitivity, which is of particular interest due to the low clinically relevant levels of exosomes or CTCs. A microfluidic platform, ExoPC D-chip, designed for isolation and detection of exosomes in human serum (just 30 μL required), discriminates healthy individuals from patients with hepatic carcinoma in <4 h (Xu et al., 2018). The chip has two differentiated areas for magnetic capture of exosomes, with capture efficiency about 3-times higher than that of a commercial kit, and downstream electrochemical detection onto an ITO electrode. Specific exosome quantification is accomplished by using an in-solution hairpin DNA probe harboring CD63 aptamer and DNAzyme sequences, which is open in the presence of CD63-positive exosomes, giving rise to a catalytic signal.

A more general detection strategy is proposed by (An et al., 2019). An aptamer is immobilized on the surface of a nanostructured electrode surface for capturing exosomes. Isothermal signal amplification by hybridization chain reaction (HCR) is used for signal amplification, by attaching an initiator DNA strand to the captured exosomes via click chemistry. The main drawback of this approach is the longer assay time without substantial gain in sensitivity.

In spite of the advantages of aptamer capture—such as the easy to disrupt aptamer-exosome binding, either by displacement with a complementary oligonucleotide or by DNA-cutting enzymes—immunoaffinity capture of exosomes is still predominant. (Huang et al., 2019) combined this strategy with an aptamer for detection (sandwich approach) so they can benefit from amplification strategies not amenable to antibodies pushing down the LOD of these elusive targets.

In sum, liquid biopsy in blood is advancing quickly toward PCR-free, extraction-free strategies for cfNA detection by means of nanostructuring, and signal amplification strategies. The latter is suitable for concatenation (cascades) but it must be kept to a minimum to be viable in POC employed by non-specialized staff. CTCs and exosome detection with aptamers is still in its infancy and needs to face clinical samples instead of the dominant spiked serum/plasma to be seriously considered for clinical usage. The availability of novel aptamers for features specifically expressed under several disease stages is the bottleneck that needs to be surpassed to boost the development of aptamer-based *in-vitro* diagnostic devices.

## DNA-Based Yellow Diagnostics

Unlike blood, urine-based liquid biopsy is completely non-invasive (home sample collection is indeed feasible), and it allows for more frequent monitoring independently of patient's health status and without restrictions in the collected specimen volume. In addition to these practical advantages favorable to point-of-care testing, the use of urine-based tests can also promote the early detection of urologic cancers (namely prostate, bladder, and kidney) due to the proximity of this fluid to the primary tumor; while blood tests tend to exhibit poorer sensitivity since vascular invasion would occur at an advanced stage of the disease (Peng et al., 2017).

Despite this promising atmosphere, just a few electrochemical DNA-based biosensors have been developed for urine-based biopsy so far, and they mainly employ spiked synthetic urine for validation. As an example, a microfluidic electrochemical device with multiplexing capability for three methylation DNA biomarkers associated with bladder cancer exhibited femtomolar level detection within 20 min, but it was only tested in an enriched urine-mimicking matrix with unknown content in other NA (Pursey et al., 2017). Unspecific protein adsorption and matrix effects appear when using patient samples with label-free techniques. In an impedimetric hybridization-based biosensor for miRNA-21 reporting fM LOD, urine was digested with proteinase K and then filtrated (Smith et al., 2017). That way, an antifouling strategy to protect the surface is unnecessary while miRNA integrity is preserved, though direct detection is not viable. Even when the access to the electrode of the redox probe is restricted to nanochannels within an aptamer-modified silica film, matrix effects preclude direct detection of the target PSA (Argoubi et al., 2018).

These rather simple approaches contrast with an aptamer-based assay for the determination of epithelial cell adhesion molecule (EpCAM), where an enzyme-free strand displacement reaction is combined with isothermal DNA amplification (Chen et al., 2018). The utility of this apta-test was evaluated in different spiked body fluids, including urine, after dilution with a suitable buffer solution to fix the pH and the ionic strength.

It is apparent that yellow diagnostics is less explored and the lower availability of well-studied tumor biomarkers is reflected in the much fewer number of approaches sampling urine. The fact that less effort is invested here delays the development of strategies to fully achieve the direct measurement.

## DNA-Based White Diagnostics

Saliva is 94–99% water, and easy to collect in large amounts (humans generate up to 1 L/day), but is poorer than blood in analytes, except for ctDNA from oral cancer (Wang et al., 2015). The advent of more sensitive techniques has boosted the interest in both this fluid and sweat (“white diagnostics”) as a source of cancer biomarkers entering from the blood.

The electrochemical technology EFIRM (Electric Field-Induced Release and Measurement), commercially exploited by Ezlife, was used to validate salivary biomarkers in oral (Wei et al., 2009) and lung (Wei et al., 2014) cancers. The innovative cycling square wave electric field method works by speeding up the target-probe interaction from 30 to 60 min to <5 min and also permits the cargo release from exosomes (Wei et al., 2013). The main advantage is the simultaneous determination of targets of a different nature, i.e., proteins and NA in the same sample but separate electrodes of a single chip without needing individual assay optimization. Saliva analysis is performed after minimal pretreatment in 10 min with a LOD of 3.9 fM for mRNA (Wei et al., 2009). The use of undiluted saliva requires proper antifouling strategies while admitting high target binding capacity as demonstrated with a hybrid nanomaterial composed of reduced graphene oxide and carboxymethylcellulose (Esteban-Fernandez de Avila et al., 2015). Discrimination of a single central mismatched TP53 sequence was possible in spiked saliva, but at relatively high concentrations.

Low detectability is claimed for DNA (12.8 fM) (Ma et al., 2018b) with exonuclease assisted target recycling. To decrease the LOD, avoidance of direct contact between biofluids and the electrode is an alternative approach. This is straightforward on magnetic beads with final MB capture on the electrode surface. Ultrasensitivity of miRNA (0.22 aM) was achieved with enzymatic signal amplification (Wang et al., 2013) but all these approaches were only tested in spiked saliva.

As an ultrafiltrated fluid, saliva has extremely low protein content and aptasensors are currently mainly directed to non-protein targets, such as drug of abuse, according to Scopus database. Nonetheless a prototype of a portable field-effect transistor based on graphene with wireless capability for remote diagnosis has been recently reported to detect a pancreatic cancer biomarker with a remarkable LOD (12 pM), though centrifugation and saliva dilution is still needed (Hao et al., 2019).

The other white fluid, sweat, has found higher applicability in sports medicine until now, because the clinical utility of tumor biomarkers needs to be fully addressed. Nonetheless, an aptamer-based assay has been reported for interleukin-6, a potential biomarker of cancer but also inflammation in artificial sweat (Kumar et al., 2016).

The potential of saliva for liquid biopsy is just finding its way accompanied by a demonstration of its clinical validity with an increasing number of biomarkers (Kaczor-Urbanowicz et al., 2019). Investment in commercial development would suggest that the interest in this fluid will grow in the next decade. On the contrary, sweat seems to be less suited for tumor biomarker detection unless a breakthrough takes places in the near future. Table 1 summarizes the main characteristics of the above commented methods.

**Table 1:** Main characteristics of selected electrochemical methods using a nucleic acid as a receptor for the detection of tumor biomarkers in biological fluids (liquid biopsy).

**Sample**	**Analyte**	**Collection/pretreatment**	**Receptor**	**Amplification strategy**	**Main features**	**Calibration range**	**LOD**	**References**
Serum	cfNA (KRAS and BRAF mutated sequences)	Unprocessed	PNA Capture probe	None	Multiplexing 15 min assay time 5 fg of isolated DNA (50 μL serum)	1 fg μL^−1^-100 pg μL^−1^	1 fg μL^−1^	Das et al., 2015
Serum	cfDNA (EGFR gene)	Unprocessed	PNA Capture probe	None	Multiplexing	–	–	Das et al., 2018
Plasma	cfDNA (KRAS G12DM)	Kit extraction	Triple-helix molecular switch	Target recycling +branched TdT	Extreme detectability Pretreatment required	0.01 fM−1 pM	2.4 aM	Wang et al., 2018
Plasma	Methylation in genomic DNA	Phenol-chloroform extraction	None (direct genomic DNA adsorption)	None	10 min analysis time 200 human samples of gDNA	–	–	Sina et al., 2018
Serum urine	Prostate cancer genetic aberrations in RNA (TMPRSS2-ERG, PCA3, or SChLAP1)	NA extraction by on-chip electric lysis + magnetic washing 10-fold serum dilution Undiluted urine	c-DNA	RT-RPA prior to detection (target amplification)	Integrated nanofluidics biochip (lysis+amplification +detection)	0–1,000 copies	50 copies	Koo et al., 2018
Serum	HER2/neu	10-fold dilution	aptamer	Enzymatic	No protein fouling	10 fM−100 pM	1 fM	Malecka et al., 2019
Serum	CEA	Dilution	aptamer	HCR	Good agreement with stablished immunoassay. Complex assay	0.0001–50 ng mL^−1^	18.2 fg mL^−1^	Liu et al., 2019
Serum	CEA	5-fold dilution	Hairpin-aptamer	Gold nanorods with multiple enzymes	Good agreement with stablished immunoassay Complex assay	5 pg mL^−1^-50 ng mL^−1^	1.5 pg mL^−1^	Wen et al., 2016
Serum	CEA and MUCIN-1	High dilution	aptamers	Au-BSA nanospheres	Good agreement with stablished immunoassay Dual detection Complex assay	0.01 pM−100 nM	3.33 fM	Ma et al., 2018a
Serum	CEA and NSE	Undiluted	aptamers	PB-PEDOT-AuNPs nanocomposites	Good agreement with stablished immunoassay	0.01–500 ng mL^−1^	2 pg mL^−1^	Wang et al., 2019b
Serum	PSA	2-fold dilution	Protein- binding aptamer (capture) + glycan-binding aptamer (detection)	Enzymatic	Potential better discrimination between cancer and benign diseases	0.66–25 ng mL^−1^	0.66 ng mL^−1^	Díaz-Fernández et al., 2019
Serum	Exosomes	Exosome isolation kit	Aptamer for detection + lipid-binding protein for capture	DNAzyme	Integrated microfluidic platform for isolation and detection 30 μL sample volume	7.6 ×10^4^-7.6 ×10^8^ particles mL^−1^	4.4 ×10^3^ particles mL^−1^	Xu et al., 2018
Serum	Exosomes	Exosome isolation kit	CD63 aptamer	HCR	General detection strategy based on click chemistry attachment of a DNA initiator onto exosome surface	112–1.12 ×10^8^ particles μL^−1^	96 particles μL^−1^	An et al., 2019
Serum (spiked)	Exosomes	Culture cell lines subjected to centrifugations + filtrations	EpCAM and CD63 Aptamers	Ti_3_C_2_ MXenes nanosheets	LOD 100 times lower than ELISA method. Possible cargo downstream analysis.	5 ×10^5^-5 ×10^9^ particles mL^−1^	1.25 ×10^4^ particles mL^−1^	Zhang et al., 2019
Plasma	Gastric cancer exosomes	Ultracentrifugation and dilution	MUC-1 aptamer	RCA	Tested in gastric cancer patients Sandwich format with a capture antibody	4.8 ×10^3^-4.8 ×10^6^ particles mL^−1^	950 particles mL^−1^	Huang et al., 2019
Whole blood	Leukemia CTCs	Culture cell line centrifuged and resuspended in buffer or blood	aptamers	AuNP array-decorated magnetic graphene nanosheet	Multiplexing Tested in leukemia patients blood	5–500 cells mL^−1^	3 cells mL^−1^	Dou et al., 2019
Serum	CTCs	Culture cell line centrifuged and resuspended in serum samples	EpCAM aptamer	None	Possible cargo downstream analysis. Reusable cytosensor (8 times) Wide linear range	30–10^6^ cells mL^−1^	10 cells/mL	Shen et al., 2016
Urine	Bladder cancer DNA markers (*E. Cad, DAPK, RARβ*)	Enriched urine-mimicking matrix	Hairpin c-DNA	None	Multiplexing 20 min assay time	10^−13^-10^−7^ M (nonlinear)	250 fM	Pursey et al., 2017
Urine	miRNA-21	Digestion with proteinase K + filtration	c-DNA probe	None	No protein fouling of electrode surface	10 fM−10 nM	20 fM^*^	Smith et al., 2017
Urine (spiked)	PSA	Unprocessed	Aptamer	None	Good storage stability (1 month at 4°C)	1–300 ng mL^−1^	280 pg mL^−1^	Argoubi et al., 2018
Urine	EpCAM	50% dilution	Aptamer	Target-driven toehold-mediated DNA recycling amplification	Regenerable sensing surface	0.1–20 ng mL^−1^	20 pg mL^−1^	Chen et al., 2018
Saliva	IL-8 mRNA	Centrifugation RNAse inhibition	c-DNA probe	Enzymatic	*In-situ* NA release by non-uniform electric field PCR free Results in 10 min Patient and control samples tested	5 fM-50 pM	3.9 fM	Wei et al., 2009
Saliva	EGFR mutations					–	–	Wei et al., 2014
Saliva	GAPDH mRNA from exosomes					–	–	Wei et al., 2013
Saliva (spiked)	P53 tumor supresor gene	No pretreatment	Hairpin c-DNA	Enzymatic	PCR free SNP selectivity Limited sensitivity	10–100 nM	2.9 nM	Esteban-Fernandez de Avila et al., 2015
Saliva (spiked)	ORAOV1	Centrifugation and 10-fold dlution	c-DNA probe	Homogeneous target recycling	PCR free Limited number of samples tested	20 fM−2 nM	12.8 fM	Ma et al., 2018b
Saliva (spiked)	hsa-miRNA-200a	Centrifugation and 100-fold dilution	c-DNA on magnetic beads	Enzymatic	PCR like sensitivity Limited number of samples tested	1 aM−10 fM	0.22 aM	Wang et al., 2013
Saliva (spiked)	IL-6	Centrifugation and 1,000-fold dilution	Anti-IL6 aptamer	None	400 s response time Portable Wifi connection	0.05–20 nM	12 pM	Hao et al., 2019

*c-DNA, complementary DNA, CEA, carcinoembryonic antigen; EGFR, endothelial growth factor receptor; EPCAM, epithelial cell adhesion molecule; GAPDH, Glyceraldehyde-3-phosphate dehydrogenase, gDNA, genomic DNA; HCR, hybridization chain reaction; IL, interleukin; OROV1, oral cancer overexpressed 1; NSE, neuron specific enolase; PB-PEDOT prussian blue-poly (3,4- ethylenedioxythiophene); PSA, prostatic specific antigen; RCA, rolling circle amplification; RT-RPA, reverse transcription recombinase polymerase amplification; TdT, terminal deoxynucleotidyl transferase*.

* *LOD higher than the lowest concentration tested in the linear plot means that the linear range was not reliably established*.

## Translation to the Clinical Practice

The creativity of researchers to combine the wide variety of nanomaterials with specifically-designed DNA probes and amplification schemes is endless. However, simplicity is a compulsory feature of POC devices, along with non-marginal improvement over current technology, to attract investors who will translate them into commercial devices and make viable the analytical validation. This step requires the analysis of a huge number of samples and is lacking in many academic works.

Meeting the strict regulations for medical use of *in-vitro* diagnostic devices is also essential in order to reach the market. Clinical validity depends on biomarker clinical usefulness. It relies on extensive years-long clinical trials that lag behind the active biosensing research field. Thus, the paradoxical scenario of developing tools for non-fully validated targets is a critical barrier for commercialization of electrochemical and other kinds of biosensors. Concurrently, low-cost, and rapid tools as biosensors can speed up the target validation.

Electrochemical DNA-biosensors are reaching the capability of detecting ctNA with minimal or no sample pretreatment but at the expense of correcting the sensor drift due to surface fouling, using disposable platforms (increasing waste) or developing smart schemes to protect the sensing layer (Campuzano et al., 2019; Yang et al., 2019). This also holds for aptasensors that use thiol-PEG (Salimian et al., 2017) or zwitterionic peptides (Cui et al., 2017) as blocking agents, as well as mixed-self assembled monolayers for subsequent covalent immobilization of the receptor (Díaz-Fernández et al., 2019). In any case, sample collection needs harmonization. As long as this is achieved, more biosensors will pass the administration scrutiny.

Microfluidic technology reduces the conventional multistep workflow so it is becoming essential in pursuing the integration of isolating rare cells and exosomes with analysis, commonly in conjunction with magnetic particles (Contreras-Naranjo et al., 2017). Though they rely on immunocapture (Chang et al., 2019), one can envision the successful use of aptamers for an identical purpose.

Companies are investing heavily in hand-held platforms with mobile-phone connectivity or smartphone-based devices to decentralize the expensive clinical analysis requiring large benchtop instrumentation and specialized staff. In tumor biomarker detection, this technology is less explored than for drug abuse analysis or other widespread disorders. It is worth noting that electrochemical detection is the choice for quantitative *in-vitro* diagnostics (Shin et al., 2018).

## Conclusions

The development of electrochemical DNA-based devices for cancer biomarkers is growing at a good pace but is somehow slower than other transduction techniques. The outstanding features they offer cannot be taken aside with the excuse of the relatively complex theoretical foundation of some smart electrochemical measurement schemes. The advances are more visible in blood testing but they must be adapted to other fluids, especially urine, with a huge potential in liquid biopsy. Innovation in format schemes is needed to meet the extreme sensitivity liquid biopsy demands but complex cascades of amplification schemes may not be the route if POC devices are in the spotlight. Otherwise, paying more attention to sample issues is desirable.

Many hybridization assays are now commercial so it is expected that their translation to a low-cost biosensor format will attract investors' attention. On the contrary, aptamers are mainly unknown by a medical community accustomed to antibodies as the reagent of choice for affinity tests. In recent years, a higher interest in them is apparent but their excellent features and versatility needs to be reinforced with reliable clinical applications. The antibody “industry” should not be threatened by aptamers. On the contrary, they should consider them as a complementary technology to fill the gap where antibodies fail, such as directing the selection to specific regions (often low immunogenic or hindered) of the targets. In clinical settings, microfluidics and biosensors with direct connectivity are already reality and these trends will coexist with future smartphone-based devices, provided that they overpass the current limitations in accuracy and reproducibility. We also envision that multiplexing capability will be crucial in future biosensors because of the superior specificity/sensitivity of combination of biomarkers in early detection of cancer.

## Author Contributions

RM-C, IP, and NS-Á conceived the work, wrote, and revised the manuscript.

### Conflict of Interest

The authors declare that the research was conducted in the absence of any commercial or financial relationships that could be construed as a potential conflict of interest.
